# Comprehensive Phytochemical Characterization and Quality Evaluation of *Taxillus chinensis* via Integrated Widely Targeted Metabolomics, HPLC Fingerprinting, and Multi-Component Quantification

**DOI:** 10.3390/metabo16070446

**Published:** 2026-06-25

**Authors:** Zhouwei Li, Hongfei Wei, Jiahui Wu, Qiyuan Yang, Jiemei Liang, Xiaoxun Wang, Li Li

**Affiliations:** 1 School of Pharmacy, Guangxi University of Chinese Medicine, Nanning 530200, China; lizhouwei2023@stu.gxtcmu.edu.cn (Z.L.); yangqiyuan2023@stu.gxtcmu.edu.cn (Q.Y.); 13590954856@163.com (J.L.); 2 Guangxi Key Laboratory of Zhuang and Yao Ethnic Medicine, Guangxi University of Chinese Medicine, Nanning 530200, China; 3 University Engineering Research Center of Development and Industrialization of Zhuang and Yao Ethnic Medicinal Materials, Guangxi University of Chinese Medicine, Nanning 530200, China; 4 The Collaborative Innovation Center of Zhuang and Yao Ethnic Medicine, Guangxi University of Chinese Medicine, Nanning 530200, China; 5 Guangxi Engineering Research Center of Ethnic Medicine Resources and Application, Guangxi University of Chinese Medicine, Nanning 530200, China

**Keywords:** *Taxillus chinensis*, widely targeted metabolomics, fingerprinting, multi-component quantification, quality evaluation

## Abstract

Background/Objectives: This study aims to establish a systematic phytochemical characterization and quality evaluation method to systematically evaluate the influence of multiple factors on the chemical composition of *Taxillus chinensis*, thereby providing a scientific basis for its development, utilization, and quality control standards. Methods: To ensure a targeted and representative metabolic screening, six representative batches covering the major geographical origins and host plants were selected for initial metabolomic profiling. An integrated analytical approach combining UPLC-MS/MS-based widely targeted metabolomics, HPLC fingerprinting, and multi-component quantitative analysis with multivariate statistical analysis was employed. Results: Significant quality variations were identified across the samples. Metabolomics results indicated that while chemical component types were qualitatively consistent across growth conditions, their contents varied significantly. Unique differential metabolites clustered according to specific geographical origins or host plants. KEGG pathway analysis revealed that geographical origin primarily regulated phenylpropanoid biosynthesis, whereas host differences mainly influenced flavonoid and monoterpenoid biosynthesis. Furthermore, HPLC fingerprinting of 20 batches demonstrated similarities greater than 0.9, with 15 common peaks determined. Based on their high relative abundance, differential significance across samples, and documented pharmacological relevance to the herb’s traditional efficacy, six bioactive components—gallic acid, catechin, epicatechin, hyperoside, isoquercitrin, and quercitrin—were identified and quantified. Notably, samples originating from Wuzhou exhibited the highest total content of these components. Consistent with PCA and HCA results, gallic acid, hyperoside, isoquercitrin, and quercitrin were identified as potential markers driving quality differences. Conclusions: This integrated approach allows for a systematic analytical screening of *Taxillus chinensis*, clarifying chemical variations caused by environmental and biological factors, and supporting the standardization and comprehensive utilization of this medicinal plant.

## 1. Introduction

*Taxillus chinensis* is the dried leafy stem and branch of *Taxillus chinensis* (DC.) Danser, a plant belonging to the genus Taxillus in the family Loranthaceae [1]. It is neutral in nature, bitter and sweet in taste, and acts on the liver and kidney meridians. It possesses efficacy in dispelling wind-dampness, tonifying the liver and kidney, strengthening tendons and bones, and calming the fetus. *Taxillus chinensis* parasitizes over 150 species of host plants across 36 families [2]. As a hemiparasitic plant, the composition and content of its internal metabolites undergo specific changes depending on the host species. Furthermore, factors such as growth environment, geographic origin, and harvest time also influence the quality and therapeutic efficacy of the medicinal material.

However, the 2025 edition of the *Chinese Pharmacopoeia* provides only singular qualitative quality control indicators and does not specify host plants. This creates uncertainty regarding whether *Taxillus chinensis* derived from different hosts or origins can be treated as the same medicinal entity, making it difficult to effectively evaluate its quality.

The variation in component composition and content presents a significant challenge for the quality control of Traditional Chinese Medicine (TCM) [3]. Metabolomics, by providing a comprehensive interpretation of complex TCM chemical compositions combined with multivariate statistical analysis, can screen for significantly differential metabolites among different batches, thereby achieving a clear distinction of source and quality. Additionally, fingerprinting and multi-component quantification, when combined with chemometrics, can rapidly evaluate the consistency of different batches and quantify key components. This facilitates a comprehensive quality evaluation that moves from the “holistic” to the “specific,” serving as a critical tool in TCM assessment. Combining these approaches to analyze chemical composition under different growth conditions provides a more scientific basis for quality evaluation and control [4,5].

Existing studies indicate that the quality of *Taxillus chinensis* is regulated by multiple factors. Parasitism on different hosts leads to differences in chemical components [6], metabolite synthesis [7], and medicinal properties [8]. Variations in the stem-to-leaf ratio also affect chemical composition [9]. Furthermore, differences in harvest time and growth environment significantly impact the accumulation of secondary metabolites [10], subsequently affecting the therapeutic quality of the herb [11]. Based on this, comprehensive analysis, hierarchical screening, and quantitative detection of active ingredients are required to satisfy the needs for quality control and development.

**Figure 1:**
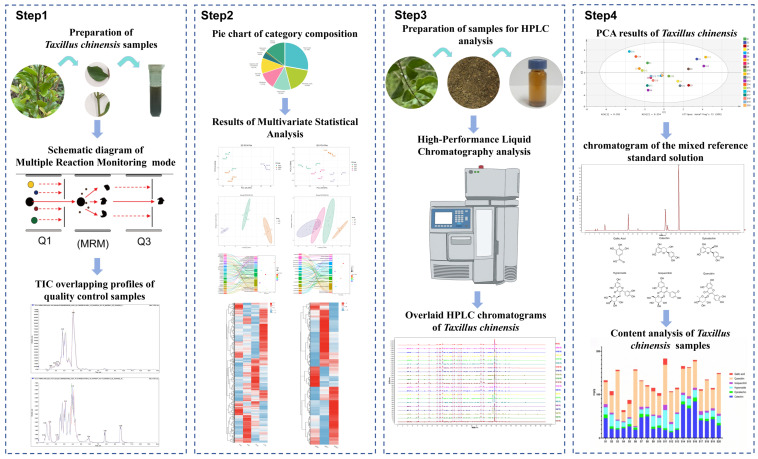
Integrated analytical workflow for the quality evaluation of *Taxillus chinensis*.

To investigate the impact of host, origin, and harvest time on the chemical composition of *Taxillus chinensis*, this study employs metabolomics, fingerprinting, and multi-component quantitative analysis combined with chemometrics. Drawing on established methodological frameworks in TCM quality evaluation, the study systematically analyzes the chemical components of *Taxillus chinensis* from different origins and hosts: Metabolomics is utilized to provide an unbiased screening of global metabolic profiles to identify differential components [12]; fingerprint similarity is used to evaluate the overall consistency of chemical characteristics to determine general quality trends [13]; and multi-component quantitative analysis determines the precise content of components to elucidate markers related to quality [14]. While widely targeted metabolomics and fingerprinting provide a comprehensive exploratory view of the complex chemical compositions, specific multi-component quantification is essential for establishing practical quality control standards. In this study, based on the broad screening of widely targeted metabolomics and the determination of common peaks in HPLC fingerprints, six core bioactive components—gallic acid, catechin, epicatechin, hyperoside, isoquercitrin, and quercitrin—were specifically selected as quantitative markers. The rationale for selecting these specific markers is based on their high relative abundance, differential significance across various samples, and highly documented pharmacological relevance to the herb’s traditional efficacy (e.g., anti-inflammatory and dispelling wind-dampness). This selection logically bridges the gap between widely targeted global profiling and targeted quality evaluation, ensuring the identified markers are both analytically representative and clinically relevant. This study aims to provide a scientific basis for establishing a more accurate and comprehensive quality evaluation method and quality control standard for *Taxillus chinensis*. The detailed experimental workflow is depicted in Figure 1.

## 2. Materials and Methods

### 2.1. Materials

#### 2.1.1. Instruments

Scientz-100F freeze dryer (Ningbo Scientz Biotechnology Co., Ltd., Ningbo, China); MM400 mixer mill (Retsch, Haan, Germany); MS105DU semi-micro analytical balance (Mettler Toledo, Greifensee, Switzerland); Chromatographic column: Agilent SB-C18 (1.8 µm, 2.1 mm × 100 mm) (Agilent Technologies, Santa Clara, CA, USA); Agilent 5 TC-C18(2) 250 × 4.6 mm (Agilent Technologies, Santa Clara, CA, USA); ExionLC™ Ultra-Performance Liquid Chromatography (UPLC) system coupled with an AB Sciex QTRAP 6500 LC/MS/MS System (AB Sciex, Framingham, MA, USA). Waters e2695 High-Performance Liquid Chromatography (HPLC) system (Waters Corporation, Milford, MA, USA); SQP electronic balance (Sartorius Scientific Instruments (Beijing) Co., Ltd., Beijing, China); JA21002B electronic balance (Shanghai Techcomp Balance Instrument Co., Ltd., Shanghai, China); KQ-500DA CNC ultrasonic cleaner (Kunshan Ultrasonic Instrument Co., Ltd., Kunshan, China); RE-2000E rotary evaporator (Zhengzhou Yarong Instrument Co., Ltd., Zhengzhou, China); DLSB-5/20B low-temperature cooling liquid circulating pump (Zhengzhou Great Wall Sci-Industry and Trade Co., Ltd., Zhengzhou, China), etc.

#### 2.1.2. Reagents

Methanol and acetonitrile were of chromatographic grade (Merck, Germany; Aladdin Biochemical Technology Co., Ltd., Shanghai, China), and formic acid was of chromatographic grade (Aladdin Biochemical Technology Co., Ltd., Shanghai, China). Gallic acid (Batch No.: C16806656, Purity: 99%, Shanghai Macklin Biochemical Co., Ltd., Shanghai, China); Catechin (Batch No.: S01HB191501, ≥98%, Shanghai Yuanye Bio-Technology Co., Ltd., Shanghai, China); Epicatechin (Batch No.: M07HB177342, ≥98%, Shanghai Yuanye Bio-Technology Co., Ltd.); Hyperoside (Batch No.: E2507566, ≥98%, Shanghai Aladdin Biochemical Technology Co., Ltd.); Isoquercitrin (Batch No.: MUST-25070722, Purity: 99.99%, Chengdu Push Bio-Technology Co., Ltd., Chengdu, China); Quercitrin (Batch No.: G2418566, ≥98%, Shanghai Aladdin Biochemical Technology Co., Ltd.); Ethyl acetate (Analytical grade, Batch No.: 250311E1, Sichuan Xilong Scientific Co., Ltd., Chengdu, China), etc.

#### 2.1.3. Plant Materials

The plant materials were identified by Associate Professor Li Li of Guangxi University of Chinese Medicine as the dried leafy stems and branches of *Taxillus chinensis* (DC.) Danser. The source information for the *Taxillus chinensis* materials is shown in Table 1. The samples used for metabolomics analysis were S1 (CSJ), S10 (NSJ), S11–S13 (WFJ, WSJ, WSO), and S20 (YSJ).

To ensure the interpretability and representativeness of the metabolomics analysis, six core samples were carefully selected using a controlled variable approach. The specific combinations of samples with “identical host (*Morus alba* L.) but different origins” (S1, S10, S12, S20) and “identical origin (Wuzhou) but different hosts (*Morus alba* L., *Liquidambar formosana* Hance, and *Pinus massoniana* Lamb.)” (S11, S12, S13) accurately decoupled the independent effects of geographical and host factors on metabolite accumulation. Furthermore, *Taxillus chinensis* parasitizing these three typical hosts all exhibit the definite traditional efficacy of “dispelling wind-dampness.” Including them highly aligns with real-world clinical applications, providing a highly representative scientific basis for elucidating their pharmacodynamic material basis.

### 2.2. Methods

#### 2.2.1. Metabolomics Study

##### Sample Preparation of *Taxillus chinensis*

Fresh plant materials were collected and immediately divided into 6 groups using 50 mL centrifuge tubes, with 3 biological replicates per group. The samples were flash-frozen in liquid nitrogen and subsequently transferred to an ultra-low temperature freezer (−80 °C) for storage. The 18 frozen samples were lyophilized and ground into powder using a mixer mill.

Fifty milligrams (50 mg) of sample powder were weighed and extracted with 1200 µL of a pre-cooled (−20 °C) extraction solution containing an internal standard. The internal standard extraction solution consisted of methanol and ultrapure water (7:3, *v*/*v*), prepared by combining 70 mL of methanol, 30 mL of ultrapure water, and 1 mL of L-2-phenylalanine internal standard stock solution (1 mg/mL, prepared by dissolving 5 mg ± 0.1 mg of L-2-phenylalanine in methanol to a final volume of 5 mL). The solution was mixed well and stored at −20 °C, with a validity period of 7 days. The sample mixture was vortexed for 30 s every 30 min for a total of 6 cycles. After centrifugation at 12,000 rpm for 3 min, the supernatant was filtered through a 0.22 µm microporous membrane and stored in a vial for UPLC-MS/MS analysis.

##### UPLC Chromatographic Conditions

Chromatographic separation was performed using an Agilent SB-C18 (2.1 mm × 100 mm, 1.8 µm) maintained at 40 °C. The mobile phase consisted of solvent A (ultrapure water with 0.1% formic acid) and solvent B (acetonitrile with 0.1% formic acid). The elution gradient was established as follows: 0–9.0 min, 5–95% B; 9.0–10.0 min, 95% B; 10.0–11.0 min, 95–5% B; 11.0–14.0 min, 5% B. The flow rate was 0.35 mL·min^−1^, and the injection volume was 2 µL.

##### Mass Spectrometry Conditions

An electrospray ionization (ESI) source was used with a source temperature of 500 °C. The spray voltage was set to 5.5 kV in positive ion mode and −4.5 kV in negative ion mode. The pressures for ion source gas I (GSI), gas II (GSII), and curtain gas (CUR) were set to 50, 60, and 25 psi, respectively. The collision-activated dissociation (CAD) parameter was set to “high.” Scanning was performed in multiple reaction monitoring (MRM) mode with nitrogen used as the collision gas, set to “medium”.

##### Data Processing and Analysis

Qualitative and quantitative analyses of metabolites in *Taxillus chinensis* from different origins and hosts were conducted using the AB SCIEX QTRAP 6500 mass spectrometry platform and the Metware in-house database. Based on the in-house database, metabolites were identified according to secondary mass spectrometry (MS/MS) spectral information. During the analysis, isotopic signals, duplicate signals containing K^+^, Na^+^, and $NH_{4}^{+}$ ions, as well as duplicate signals of fragment ions derived from other larger molecules, were removed. The exclusion of isotopic, redundant, and interfering signals during the qualitative analysis was performed to enhance identification accuracy. Metabolite quantification was carried out using the multiple reaction monitoring (MRM) mode of a triple quadrupole mass spectrometer. After acquiring the metabolite mass spectrometry data from various samples, the chromatographic peak areas for each sample were integrated, and the mass spectral peaks of the same metabolite across different samples were subjected to integration correction to achieve precise quantification.

### 2.3. Fingerprint Analysis and Multi-Component Quantitative Analysis

#### 2.3.1. Preparation of Solutions

##### Preparation of Reference Standard Solutions

Appropriate amounts of each reference standard were accurately weighed and dissolved in 50% methanol under ultrasonication to prepare single standard stock solutions with a concentration of 1 mg/mL. Subsequently, 1.0 mL of each single standard solution was accurately transferred into a 10 mL volumetric flask, diluted to volume with 50% methanol, and mixed thoroughly. The mixture was filtered through a 0.45 μm microporous membrane to obtain the mixed reference standard solution.

##### Preparation of Test Solutions

Samples of *Taxillus chinensis* from different batches were crushed and passed through a 60-mesh sieve. Ten grams (10 g) of the sample powder were extracted twice under reflux with 50% methanol (using 10-fold and 8-fold volumes, respectively) for 2 h each time. The residues were discarded, and the filtrates were combined, filtered under vacuum, and concentrated using a rotary evaporator. An appropriate amount of hot water was added to the concentrate, followed by extraction with ethyl acetate. The ethyl acetate fraction was evaporated to dryness to obtain the *Taxillus chinensis* extract powder. Two milligrams (2 mg) of this extract powder were accurately weighed into a 2 mL volumetric flask, dissolved and diluted to the mark with 50% methanol, shaken well to ensure complete dissolution, and filtered through a 0.45 μm microporous membrane to obtain the test solution.

#### 2.3.2. HPLC Chromatographic Conditions

Chromatographic separation was performed on an Agilent 5 TC-C18(2) column (250 mm × 4.6 mm). The mobile phase consisted of acetonitrile (A) and 0.1% aqueous formic acid (B). The gradient elution program was as follows: 0–15 min, 95–85% B; 15–30 min, 85–80% B; 30–35 min, 80–75% B; 35–50 min, 75–73% B; 50–60 min, 73–70% B. The column temperature was maintained at 30 °C, the flow rate was 0.8 mL/min, and the injection volume was 10.0 μL.

## 3. Results

### 3.1. Visual Analysis of Total Ion Chromatograms

**Figure 2:**
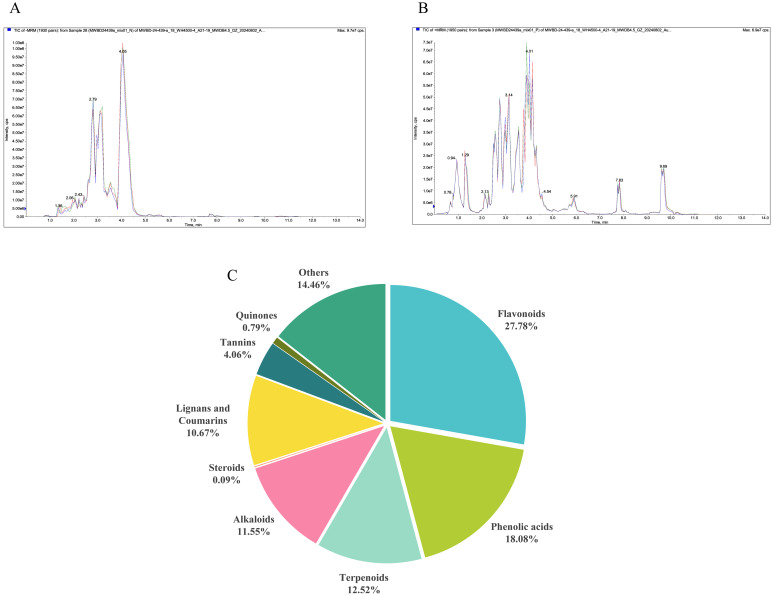
TIC overlapping profiles of quality control samples. (**A**) negative ion mode; (**B**) positive ion mode; (**C**) pie chart of category composition of *Taxillus chinensis.*

Total ion chromatogram (TIC) overlay plots of mixed quality control (QC) samples were obtained by analyzing the metabolomics of *Taxillus chinensis* from different origins and hosts using UPLC-MS/MS. The TIC results of the QC samples in both negative (Figure 2A) and positive (Figure 2B) ion modes exhibited a high degree of curve overlap. The consistency in retention times and peak intensities indicates excellent signal stability during mass spectrometric detection of the same sample at different times, confirming the reliability of the data.

The pie chart illustrating the chemical classification composition of *Taxillus chinensis* from different origins and hosts is shown in Figure 2C. A total of 1134 secondary metabolites were identified, comprising 315 flavonoids (27.78%), 205 phenolic acids (18.08%), 9 quinones (0.79%), 121 lignans and coumarins (10.67%), 131 alkaloids (11.55%), 142 terpenoids (12.52%), 1 steroid (0.09%), 46 tannins (4.06%), and 164 other compounds (14.46%).

### 3.2. Results of Multivariate Statistical Analysis

Principal Component Analysis (PCA) was performed to evaluate the differences among *Taxillus chinensis* samples based on host and origin. The analysis included samples from Wuzhou parasitizing *Morus* (WSJ), *Liquidambar* (WFJ), and *Pinus* (WSO), as well as *Morus*-parasitizing samples from Chongzuo (CSJ), Nanning (NSJ), and Yulin (YSJ).

**Figure 3:**
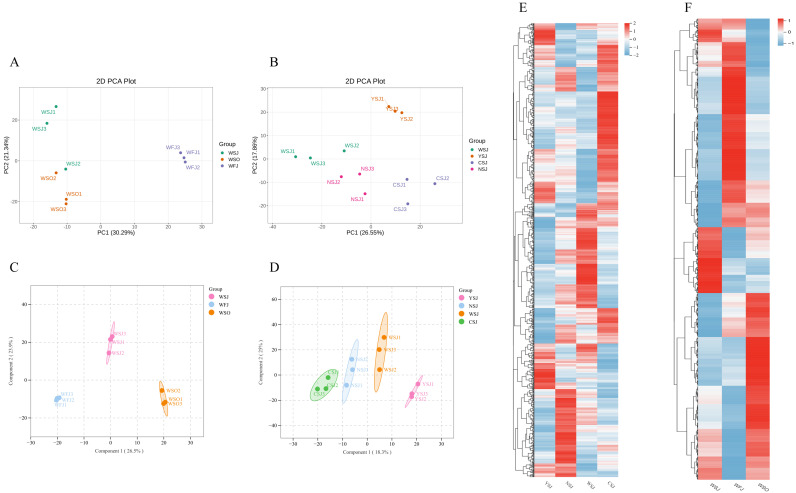
(**A**) PCA results of WSJ vs. WFJ vs. WSO; (**B**) PCA results of YSJ vs. NSJ vs. WSJ vs. CSJ; (**C**) OPLS-DA score plots for the WSJ vs. WFJ vs. WSO; (**D**) OPLS-DA score plots for the YSJ vs. NSJ vs. WSJ vs. CSJ. (**E**) Cluster heatmap of the YSJ vs. NSJ vs. WSJ vs. CSJ; (**F**) cluster heatmap of the WSJ vs. WFJ vs. WSO.

Among samples from the same origin (Wuzhou), distinct separation trends were observed. While *Morus* (WSJ) and *Pinus* (WSO) samples showed some degree of separation, both clustered significantly apart from the *Liquidambar* (WFJ) samples (Figure 3A). In contrast, samples parasitizing the same host (*Morus*) but collected from different origins exhibited minimal separation (Figure 3B). These results suggest that the host plant exerts a more significant influence on the secondary metabolite profile of *Taxillus chinensis* than the geographical origin.

To identify differential metabolites, Orthogonal Partial Least Squares Discriminant Analysis (OPLS-DA) was conducted using screening criteria of Variable Importance in Projection (VIP) ≥ 1 and a Fold Change (FC) ≥ 2 or ≤0.5. The results confirmed that both host and origin significantly impact the metabolic profile. For details on OPLS-DA analysis, refer to the Supplementary Materials.

For samples hosted by *Morus alba* L., comparisons against the Wuzhou (WSJ) group revealed 301, 180, and 283 differential metabolites in Chongzuo, Nanning, and Yulin, respectively; meanwhile, inter-origin pairwise comparisons identified 192 differential metabolites between Chongzuo and Yulin, 221 between Nanning and Yulin, and 187 between Nanning and Chongzuo. For samples originating from Wuzhou, *Liquidambar formosana* Hance samples (WFJ) showed significant differences, with 261 and 236 differential metabolites identified when compared to *Morus alba* L. and *Pinus massoniana* Lamb. samples, respectively. The comparison between *Morus alba* and *Pinus massoniana* hosts revealed 115 differential metabolites. For more detailed data on differential metabolites, please refer to Supplementary Tables S1 and S2.

**Figure 4:**
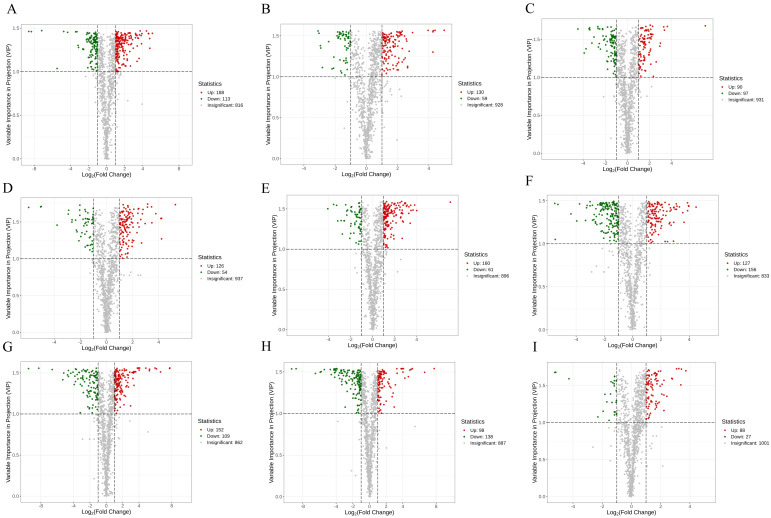
Results of volcano plots for (**A**) CSJ vs. WSJ, (**B**) CSJ vs. YSJ, (**C**) NSJ vs. CSJ, (**D**) NSJ vs. WSJ, (**E**) NSJ vs. YSJ, (**F**) YSJ vs. WSJ, (**G**) WFJ vs. WSJ, (**H**) WFJ vs. WSO, and (**I**) WSO vs. WSJ.

The primary differences among samples were observed in flavonoids and phenolic acids. Tables S3–S11 (please refer to Supplementary) list the top 10 differentially expressed metabolites. Compared with samples from different origins (CSJ, YSJ, and NSJ), WSJ exhibited distinct differences in secondary metabolite profiles. In terms of flavonoids, WSJ showed 57, 44, and 20 upregulated metabolites and 29, 18, and 14 downregulated metabolites relative to CSJ, YSJ, and NSJ, respectively. For phenolic acids, WSJ displayed 24, 46, and 13 upregulated metabolites compared with CSJ, YSJ, and NSJ, respectively, while an additional 36, 28, and 27 phenolic acid metabolites were also identified as upregulated in these same comparisons. Compared with different host samples (WFJ and WSO), WSJ exhibited clear differences in metabolite accumulation. In the flavonoid category, WSJ showed upregulation of 32 and 11 metabolites and downregulation of 38 and 27 metabolites relative to WFJ and WSO, respectively. For phenolic acids, WSJ displayed 28 and 7 upregulated metabolites, along with 20 and 14 downregulated metabolites, when compared with WFJ and WSO respectively (detailed in Figure 4).

Overall, WSJ samples possessed a higher abundance of dominant flavonoid metabolites compared to samples from other origins. However, WSJ contained fewer dominant flavonoids when compared to samples parasitizing different hosts (*Liquidambar formosana* and *Pinus massoniana*). Hierarchical Cluster Analysis (HCA) (Figure 3E,F) visually confirmed that *Taxillus chinensis* samples from each specific origin or host cluster with unique differential metabolites.

### 3.3. KEGG Pathway Analysis of Differential Metabolites

**Figure 5:**
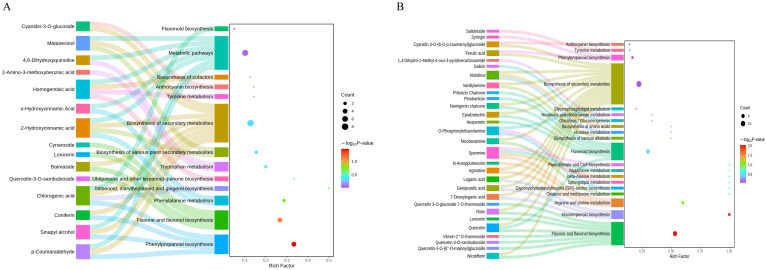
KEGG Enrichment Analysis of Differentially Expressed Metabolites in WSJ vs. WFJ vs. WSO (**A**) and WSJ vs. YSJ vs. CSJ vs. NSJ (**B**).

The differential metabolites identified in *Taxillus chinensis* from different geographical origins and hosts were mapped to the KEGG database; the results are presented in Figure 5.

The differential metabolites associated with different origins were mainly significantly enriched in the “Phenylpropanoid biosynthesis” pathway (*p* < 0.05). Meanwhile, the differential metabolites associated with different hosts were mainly significantly enriched in the “Flavonoid biosynthesis” and “Monoterpenoid biosynthesis” pathways (*p* < 0.05).

These findings indicate that origin and host act as distinct influencing factors, regulating the synthesis and accumulation of secondary metabolites in *Taxillus chinensis* through different physiological and ecological mechanisms.

### 3.4. Establishment and Analysis of Chromatographic Fingerprints

#### 3.4.1. Method Validation

##### Precision Test

The sample solution of *Taxillus chinensis* was analyzed under the chromatographic conditions described in Section 2.3.2. The solution was injected six consecutive times. Using Peak 14 (quercitrin) as the reference peak, the Relative Standard Deviations (RSDs) of the relative retention times (RRT) and relative peak areas (RPA) of the common peaks were calculated. The RSDs ranged from 0.02% to 0.15% for RRT and from 0.33% to 2.94% for RPA, indicating that the instrument precision is good.

##### Stability Test

The sample solution was analyzed at 0, 2, 4, 8, 16, and 24 h under the chromatographic conditions described in Section 2.3.2. Using Peak 14 (quercitrin) as the reference peak, the RSDs of the RRT and RPA for the common peaks were calculated. The results showed that the RRT RSDs ranged from 0.02% to 0.36% and the RPA RSDs ranged from 0.23% to 2.98%, indicating that the sample solution remains stable within 24 h.

##### Repeatability Test

Six samples from the same batch (S15) were prepared in parallel and analyzed under the chromatographic conditions described in Section 2.3.2. Using Peak 14 (quercitrin) as the reference peak, the RSDs of the RRT and RPA for the common peaks were calculated. The RSDs ranged from 0.02% to 0.12% for RRT and from 0.22% to 2.77% for RPA, demonstrating good method repeatability.

#### 3.4.2. Fingerprint Establishment and Similarity Evaluation

**Figure 6:**
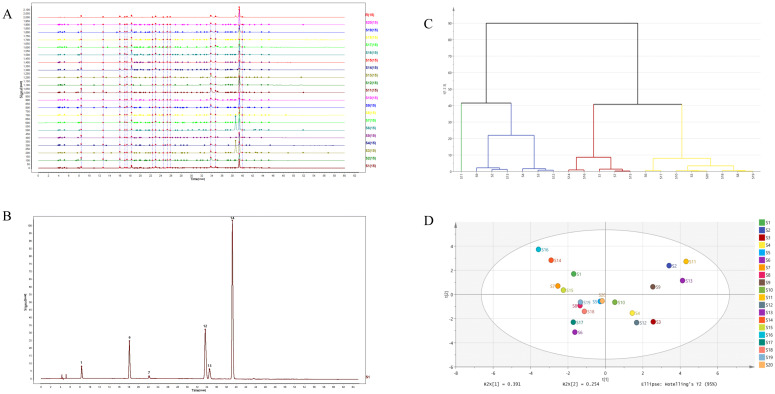
(**A**) Superimposed HPLC chromatograms of 20 batches of *Taxillus chinensis* (S1–S20) and the control fingerprint (R). (**B**) HPLC chromatogram of the mixed reference standard solution: 1. Gallic acid; 6. Catechin; 7. Epicatechin; 12. Hyperoside; 13. Isoquercitrin; 14. Quercitrin. (**C**) HCA genealogy tree diagram. (**D**) PCA results of 20 batches of *Taxillus chinensis.*

The chromatographic fingerprints for 20 batches of *Taxillus chinensis* were established by analyzing sample solutions under the conditions described in Section 2.3.2. and importing the data into the “Similarity Evaluation System for Chromatographic Fingerprint of Traditional Chinese Medicine (2012 Edition).” Using sample S15 as the reference spectrum with a time width of 0.1 min and multi-point correction, superimposed and control fingerprints were generated, revealing 15 common peaks in Figure 6A. By comparing the retention times with reference standards, six characteristic peaks were identified as gallic acid (peak 1), catechin (peak 6), epicatechin (peak 7), hyperoside (peak 12), isoquercitrin (peak 13), and quercitrin (peak 14), as detailed in Figure 6B. The similarity evaluation indicated that all 20 batches shared a similarity of greater than 0.9 with the control fingerprint, for more detailed information on the similarity of the *Taxillus chinensis* samples, please refer to Supplementary Table S12. This high degree of similarity demonstrates that *Taxillus chinensis* samples from different origins and hosts maintain relatively stable quality and good consistency, confirming that the established fingerprinting method is effective for quality control.

#### 3.4.3. Hierarchical Cluster Analysis (HCA)

The peak areas of the 15 common peaks from the *Taxillus chinensis* samples were imported into SIMCA 14.1 software. Following data standardization, hierarchical cluster analysis was performed, and the results are shown in Figure 6C. When the clustering distance was defined as 50, the 20 samples were clearly divided into two major clusters. Specifically, samples S11, S9, S2, S13, S4, S3, and S12 clustered into the first major group (left branch), whereas samples S14, S16, S7, S1, S10, S5, S20, S18, S8, S19, S15, S6, and S17 clustered into the second major group. This indicates that, regardless of differences in hosts or geographical origins, the quality of the *Taxillus chinensis* samples exhibits a certain degree of similarity.

#### 3.4.4. Principal Component Analysis (PCA)

Using the peak areas of the common peaks as variables, PCA was performed on the *Taxillus chinensis* samples from different geographical origins using SPSS 26.0 and SIMCA 14.1 software. The results are presented in Figure 6D and Table 2 and Table 3. Based on the criterion of eigenvalues > 1, four principal components were extracted, yielding a cumulative variance contribution rate of 85.603%. Principal component 1 mainly reflected the information from chromatographic peaks 1 (gallic acid), 4, 10, and 11; principal component 2 mainly reflected the information from peak 8; principal component 3 mainly reflected the information from peaks 12 (hyperoside) and 14 (quercitrin); and principal component 4 mainly reflected the information from peak 13 (isoquercitrin). These results indicate that the variations in the quality of *Taxillus chinensis* are attributed to multiple components.

### 3.5. Determination of Component Content

#### 3.5.1. Linearity Study

Appropriate amounts of reference standards for gallic acid, catechin, epicatechin, hyperoside, isoquercitrin, and quercitrin were accurately weighed. These were dissolved in 50% methanol to prepare stock solutions with mass concentrations of 20.0, 153.0, 30.0, 62.0, 45.0, and 246.0 μg/mL, respectively. The stock solutions were serially diluted with 50% methanol. The analysis was performed according to the chromatographic conditions described in Section 2.3.2. and the peak areas were recorded. Standard curves were constructed by plotting the mass concentration (x) against the peak area (Y). The results demonstrated excellent linearity for all six analytes within the investigated concentration ranges, with correlation coefficients (R^2^) exceeding 0.9991 for all compounds. The specific regression equations, correlation coefficients, and linear ranges (μg/mL) were determined as follows: for gallic acid, Y = 30,959,913.77x + 164.99 (R^2^ = 0.9993, 0.42–20.0); for catechin, Y = 6,851,251.41x − 14,150.77 (R^2^ = 0.9999, 10.2–153.0); for epicatechin, Y = 7,373,631.12x − 5763.17 (R^2^ = 0.9995, 2.0–30.0); for hyperoside, Y = 23,969,810.33x − 24,660.95 (R^2^ = 0.9991, 1.0–62.0); for isoquercitrin, Y = 33,697,619.18x − 18,167.05 (R^2^ = 0.9998, 1.0–45.0); and for quercitrin, Y = 23,105,594.58x − 62,274.30 (R^2^ = 0.9996, 16.4–246.0).

#### 3.5.2. Precision Test

The mixed reference standard solution was injected six times consecutively under the chromatographic conditions described in Section 2.3.2. The Relative Standard Deviations (RSDs) of the peak areas for the six components were calculated to be 0.98%, 0.40%, 1.80%, 0.67%, 1.03%, and 0.24%, respectively, indicating good instrument precision.

#### 3.5.3. Stability Test

The *Taxillus chinensis* sample solution was analyzed under the chromatographic conditions described in Section 2.3.2. at 0, 2, 4, 8, 16, and 24 h after preparation at room temperature. The RSDs of the peak areas for the six components were 2.51%, 0.53%, 1.39%, 1.41%, 1.45%, and 0.56%, respectively, indicating that the sample solution remains stable within 24 h.

#### 3.5.4. Repeatability Test

Six samples from the same batch of *Taxillus chinensis* were prepared in parallel and analyzed under the chromatographic conditions described in Section 2.3.2. The RSDs were 1.52%, 0.33%, 2.29%, 1.70%, 1.46%, and 0.38%, respectively, demonstrating good method repeatability.

#### 3.5.5. Recovery Test

Six samples of *Taxillus chinensis* with known component contents (approximately 1.0 g each) were accurately weighed and spiked with the respective reference standards. The samples were analyzed under the chromatographic conditions described in Section 2.3.2. The results showed that the average recoveries for gallic acid, catechin, epicatechin, hyperoside, isoquercitrin, and quercitrin were 101.12%, 102.57%, 99.19%, 97.98%, 97.36%, and 102.68%, respectively. The RSDs were 0.99%, 0.22%, 1.93%, 1.52%, 1.07%, and 1.05%, respectively, indicating good accuracy of the method. For more detailed information on recovery test, please refer to Supplementary Table S13.

#### 3.5.6. Content Determination of Samples

**Figure 7:**
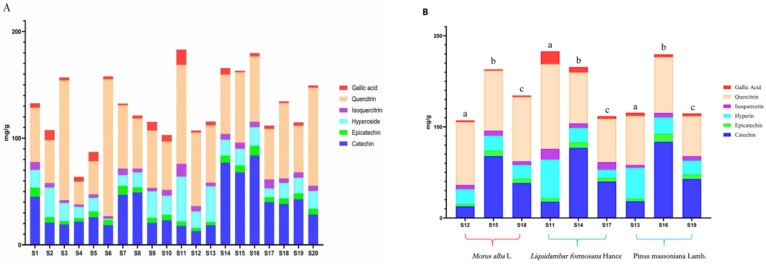
Content analysis of *Taxillus chinensis* samples. (**A**) Bar chart of content determination for all samples. (**B**) Comparison of the contents of six marker components in *Taxillus chinensis* from Wuzhou across different hosts and harvest seasons. Note: a: harvested in June 2024; b: harvested in November 2024; c: harvested in April 2025.

Twenty batches of *Taxillus chinensis* samples were prepared according to the method described in Preparation of Test Solutions Section and analyzed under the chromatographic conditions described in Section 2.3.2. The peak areas were recorded, and the contents of the six components were calculated using the standard curve method. The results are shown in Supplementary Table S14 and Figure 7.

The contents of gallic acid, catechin, epicatechin, hyperoside, isoquercitrin, and quercitrin in the 20 batches ranged from 1.33–14.13, 12.88–83.69, 2.80–9.10, 1.23–42.13, 2.01–11.85, and 21.62–128.45 μg/mL, respectively. These results indicate significant variation in the content of these components among different batches.

Sample S11 exhibited the highest content of these six components, followed by S16, S14, and S15. These specific samples were all collected from Wuzhou and utilized *Liquidambar formosana*, *Pinus massoniana*, and *Morus alba* as host plants. Based on the total content of the six components, the *Taxillus chinensis* samples from Wuzhou were significantly superior to those from other regions. This finding corroborates the scientific basis of the traditional view that “*Taxillus chinensis* is a geo-authentic herb of Guangxi, with those produced in Wuzhou being the best [15,16].” This superiority may be attributed to the unique environmental conditions in Wuzhou, which are likely more conducive to the accumulation of active ingredients.

Furthermore, a multi-factor analysis of variance (test of between-subjects effects) was employed to investigate the effects of host, origin, and harvest factors on the content of the six target components. As shown in Table 4, the results indicated that different external factors exerted varying effects on the accumulation of these components. Specifically, host and origin had a significant impact on the quercitrin content (host: *F* = 11.534, *p* = 0.003; origin: *F* = 7.333, *p* = 0.010). The harvest factor played a major role in the contents of catechins and certain flavonoids, with its effect on the catechin content reaching a highly significant level (*F* = 50.892, *p* < 0.001). It also significantly influenced the contents of epicatechin (*F* = 9.191, *p* = 0.011) and hyperoside (*F* = 5.541, *p* = 0.036). In contrast, the contents of isoquercitrin and gallic acid showed no statistically significant differences under various host, origin, and harvest conditions (*p* > 0.05).

## 4. Discussion

### 4.1. Investigation of Preparation Methods for Sample Solution

Drawing on previous literature and the preliminary stage of the experiment, ultrasonic extraction and reflux extraction methods were compared. Additionally, different extraction solvents (water, ethanol, 60% ethanol, and 50% methanol), flow rates (0.8, 1.0, and 1.2 mL/min), and the stem-to-leaf ratios of the medicinal powder were investigated. Using the number of chromatographic peaks, peak shape, and resolution as practical indicators, heat reflux extraction using 50% methanol was selected to ensure efficient and stable analysis. The finalized pretreatment method for the sample solution involved reflux extraction twice in a water bath with 10 times and 8 times the volume of 50% methanol, respectively.

### 4.2. Investigation of Chromatographic Conditions

During the preliminary screening of HPLC conditions, organic phases (methanol, acetonitrile) and aqueous phases (water, 0.1% formic acid solution, 1% formic acid solution, 0.1% phosphoric acid solution, and 1% phosphoric acid solution) were investigated. Parameters including flow rate, elution gradient, and detection wavelengths (230, 254, and 270 nm) were also evaluated. The conditions described above were finally selected as they provided comprehensive chromatogram information with minimal impurity interference. The overall peak shape, peak count, theoretical plate number, and resolution were favorable, making them suitable for the fingerprint study of *Taxillus chinensis*.

### 4.3. Analysis of Results

Historically, *Taxillus chinensis* was defined broadly as “parasitic plants on mulberry trees” without specific species identification. Currently, while the parasite species is defined as *Taxillus chinensis*, the host tree species is not restricted [17]. However, quality control still lacks quantitative detection of active ingredients, which is insufficient for the development and utilization of *Taxillus chinensis*. The objective of this study was to determine where the quality differences lie among samples from different growth environments and to identify indicator components suitable for quality evaluation.

This study established a quality evaluation method combining metabolomics, HPLC fingerprinting, and multi-component quantitative analysis for the first time. Metabolomics analysis identified a total of 1134 secondary metabolites from *Taxillus chinensis* of different origins and hosts, mainly including flavonoids, phenolic acids, alkaloids, lignans, and coumarins. Multivariate statistical analysis revealed significant differences in metabolites among the six batches, with a clear separation trend based on different origins and hosts. The differential metabolites between batches were primarily flavonoids and phenolic acids. Further KEGG analysis revealed that geographical origin primarily reshaped the ‘phenylpropanoid biosynthesis’ pathway (Figure 5A). Wuzhou’s unique abiotic stressors (e.g., intense radiation and specific soil properties) likely activate stress-responsive transcription factors, promoting the accumulation of defensive phenylpropanoids (p-coumaraldehyde, coniferin, sinapyl alcohol) as carbon skeletons for downstream flavonoids [18]. Furthermore, host plants profoundly sculpt the parasite’s pharmacological properties and metabolism via haustoria-transmitted signals [19,20]. Literature suggests that these exogenous molecules may differentially regulate rate-limiting enzymes (e.g., PAL, CHS, CHI) at the transcriptional level, which would explain the observed redirection of carbon flux toward specific flavonoid branches and the resulting chemotype differentiation [20]. Finally, PCA and HCA of the HPLC fingerprints revealed distinct seasonal clustering (Figure 6C,D). This pattern can be explained by the growth-differentiation balance hypothesis (GDBH) [21]. During active summer growth, carbon flux is prioritized for primary metabolism. However, as vegetative growth decelerates in late autumn and winter, surplus primary carbon intermediates are redirected into the shikimate pathway. This reallocation accelerates the downstream biosynthesis of phenylpropanoids and flavonoids to enhance defense against winter abiotic stresses. This mechanism is strongly supported by our multi-factor ANOVA results (Table 4), which demonstrate that harvest time significantly drives the accumulation of catechins (*F* = 50.892, *p* < 0.001), epicatechin (*p* = 0.011), and hyperoside (*p* = 0.036). Existing studies have confirmed that flavonoids and phenolic acids are the main bioactive substances responsible for the “dispelling wind and removing dampness” efficacy of *Taxillus chinensis* [22,23]. Significant differences in key active ingredients likely lead to distinctions in pharmacology or therapeutic characteristics among samples from different sources. Future research will focus on efficacy correlation analysis to clarify the biological impact of origin and host on quality. Furthermore, our metabolomics analysis detected 1-deoxynojirimycin (DNJ) in *Taxillus chinensis* parasitic on *Pinus massoniana*. Current studies indicate that DNJ is generally characterized as a specific marker derived exclusively from *Morus alba* hosts through direct substance transfer [24]. This unexpected detection in a non-mulberry host warrants further investigation, suggesting that *Taxillus chinensis* might either possess an autonomous endogenous biosynthetic pathway for DNJ or that alternative mechanisms govern its accumulation. Therefore, whether DNJ can be rigorously utilized as an exclusive characteristic marker to distinguish mulberry-hosted *Taxillus chinensis* requires further validation.

By constructing fingerprints for 20 batches of *Taxillus chinensis*, 15 common peaks were marked, and 6 common peaks were successfully identified: gallic acid, catechin, epicatechin, hyperoside, isoquercitrin, and quercitrin. Among them, gallic acid is a phenolic acid, while the others are flavonoids. Quercitrin is a core component for the anti-rheumatic effect of *Taxillus chinensis* [25]; besides significant anti-inflammatory and hepatoprotective effects [26], it can improve bone metabolism diseases by promoting osteoblast differentiation and inhibiting osteoclast formation [27]. Isoquercitrin possesses antiviral [28] and anticancer [29] properties. Gallic acid has antioxidant, anti-inflammatory, and glycolipid metabolism-improving effects [30]. Catechins exhibit anti-aging, anti-inflammatory, anticancer, and metabolic regulation effects [31]. Epicatechin, a stereoisomer of catechin, is particularly notable for its neuroprotection, muscle atrophy prevention, metabolic disease management, and vascular function improvement due to its high absorbability [32,33,34,35]. Hyperoside has pharmacological effects such as anti-inflammatory, antioxidant, anti-tumor, and wound healing properties [36]. The six components identified in the fingerprint are all active ingredients with good pharmacological activity, suggesting that *Taxillus chinensis* from different origins and hosts exerts its effects through the combined action of these components.

In the similarity analysis, all 20 batches exhibited a similarity above 0.9, indicating excellent overall consistency with profound biological significance. Specifically, this high similarity demonstrates that despite significant differences in the content of certain compounds driven by diverse host plants and geographical origins, the core macroscopic chemotype and foundational biosynthetic pathways of *Taxillus chinensis* remain genetically conserved. Ultimately, this overall stability of the chemical skeleton provides a robust material basis for its uniform traditional clinical efficacy (e.g., dispelling wind-dampness) across varying growth environments. Combining chemical pattern recognition to analyze the differences in common peaks, Hierarchical Cluster Analysis (HCA) and Principal Component Analysis (PCA) based on the peak areas of the 15 common peaks divided the 20 batches into two categories. One category (Left Cluster) included S11, S9, S2, S13, S4, S3, and S12; the other category (Right Cluster) included S14, S16, S7, S1, S10, S5, S20, S18, S8, S19, S15, S6, and S17. Comparing this with origin and host information, the clustering results were not entirely consistent with the distribution of origin or host; samples from the same origin or host did not necessarily cluster together, while samples from different origins and hosts could cluster together. However, the “Left Cluster” samples were all harvested in June 2024, while the majority of the “Right Cluster” samples were harvested after November 2024. This result indicates that the harvest season is also an important factor affecting the quality of *Taxillus chinensis*. Combined with the content determination results, samples harvested in November from Wuzhou, parasitic on *Morus alba*, *Pinus massoniana,* and *Liquidambar formosana*, showed higher overall quality, with those parasitic on *Liquidambar formosana* being the best.

## 5. Conclusions

The multi-component quantitative analysis method combining metabolomics and fingerprinting established in this study demonstrates that quercitrin, isoquercitrin, hyperoside, epicatechin, catechin, and gallic acid are closely correlated with the quality of *Taxillus chinensis*, and can serve as marker components for its quality variations. This method enables a comprehensive and systematic evaluation of the quality differences in *Taxillus chinensis*, providing a scientific basis for its quality control, the screening of superior germplasm, and its development and utilization. However, the current study still has certain limitations. First, the variables of host plant, geographical origin, and harvest season were coupled in the sampling strategy. Future studies employing strictly controlled, multi-factorial orthogonal experimental designs are necessary to comprehensively decouple these confounding variables and fully elucidate their independent impacts on the quality of *Taxillus chinensis*. Second, this study only utilized a single-solvent extraction system (aqueous methanol). Although this mature solvent system can efficiently extract the vast majority of secondary metabolites, it may under-extract certain highly non-polar or structurally unique components, thereby limiting the comprehensive characterization of the chemical composition of the entire plant matrix. Future studies should consider employing a multi-solvent combined extraction strategy to obtain more complete chemical composition information and more comprehensively reveal the overall chemical characteristics of *Taxillus chinensis*.

## Figures and Tables

**Table 1:** Sample Source Information of *Taxillus chinensis.*

Number	Location	Host Plant	Collection Date
S1 *	Ningming County, Chongzuo, Guangxi	*Morus alba* L.	June 2024
S2	Ningming County, Chongzuo, Guangxi	*Liquidambar formosana* Hance	June 2024
S3	Ertang Township, Pingle County, Guilin, Guangxi	*Morus alba* L.	June 2024
S4	Huatong Town, Jingxi, Guangxi	*Liquidambar formosana* Hance	June 2024
S5	Dawan Town, Jingxi, Guangxi	*Pinus massoniana* Lamb.	June 2024
S6	Dawan Town, Jingxi, Guangxi	*Citrus maxima* (Burm.) Merr.	May 2025
S7	Dawan Town, Jingxi, Guangxi	*Crataegus pinnatifida* Bunge.	May 2025
S8	Jinxiu Town, Jinxiu Yao Autonomous County, Laibin, Guangxi	*Morus alba* L.	January 2025
S9	Jiangnan District, Nanning, Guangxi	*Morus alba* L.	July 2024
S10 *	Guangxi Medicinal Botanical Garden, Xingning District, Nanning	*Morus alba* L.	June 2024
S11 *	Anping Town, Cenxi, Wuzhou, Guangxi	*Liquidambar formosana* Hance	June 2024
S12 *	Anping Town, Cenxi, Wuzhou, Guangxi	*Morus alba* L.	June 2024
S13 *	Anping Town, Cenxi, Wuzhou, Guangxi	*Pinus massoniana* Lamb.	June 2024
S14	Anping Town, Cenxi, Wuzhou, Guangxi	*Liquidambar formosana* Hance	November 2024
S15	Anping Town, Cenxi, Wuzhou, Guangxi	*Morus alba* L.	November 2024
S16	Anping Town, Cenxi, Wuzhou, Guangxi	*Pinus massoniana* Lamb.	November 2024
S17	Anping Town, Cenxi, Wuzhou, Guangxi	*Liquidambar formosana* Hance	April 2025
S18	Anping Town, Cenxi, Wuzhou, Guangxi	*Morus alba* L.	April 2025
S19	Anping Town, Cenxi, Wuzhou, Guangxi	*Pinus massoniana* Lamb.	April 2025
S20 *	Shinan Town, Xingye County, Yulin	*Morus alba* L.	June 2024

Note: Samples marked with * were used for the metabolomics study.

**Table 2:** Eigenvalues and variance contribution rates of principal components.

Description	Eigenvalue	Proportion of Variance	Cumulative Proportion
1	5.864	39.093	39.093
2	3.815	25.436	64.53
3	2.059	13.729	78.259
4	1.102	7.344	85.603

**Table 3:** Principal component factor loading matrix.

Peak Number	Component Matrix			
	1	2	3	4
1	0.835	0.364	0.146	−0.259
5	−0.795	0.481	−0.084	0.052
3	−0.793	0.488	0.091	−0.019
4	0.789	0.321	−0.334	0.16
10	0.764	0.296	−0.355	0.304
11	0.73	0.591	0.201	0.09
2	0.725	0.501	−0.314	0.048
6	−0.718	0.572	0.008	0.049
9	−0.63	0.537	−0.206	0.112
0	0.547	0.482	0.395	−0.421
8	0.066	0.825	−0.387	0.141
7	−0.63	0.671	−0.146	−0.046
12	−0.048	0.355	0.772	−0.071
14	0.15	0.51	0.703	0.212
13	0.059	−0.242	0.432	0.799

**Table 4:** Results of multi-factor analysis of variance for the contents of six target components affected by host, origin, and harvest factors.

Dependent Variable	Host *F*	Host *p*	Origin *F*	Origin *p*	Harvest *F*	Harvest *p*
Quercitrin	11.534	0.003 **	7.333	0.010 *	1.588	0.27
Hyperoside	1.33	0.347	2.17	0.171	5.541	0.036 *
Catechin	2.964	0.1	1.643	0.265	50.892	<0.001 ***
Isoquercitrin	0.793	0.566	0.778	0.595	0.211	0.814
Epicatechin	2.036	0.193	3.046	0.089	9.191	0.011 *
Gallic acid	1.358	0.339	0.505	0.765	1.278	0.336

Note: * *p* < 0.05; ** *p* < 0.01; *** *p* < 0.001.

## Data Availability

The data supporting this study are available within the article and its Supplementary Materials, or upon reasonable request from the corresponding author. Detailed information is provided as follows: Orthogonal Partial Least Squares Discriminant Analysis (OPLS-DA): The OPLS-DA model validation plots and permutation test score plots for metabolomics are documented in the Supplementary File named “OPLS-DA analysis” and are referenced in the section “Results of Multivariate Statistical Analysis” (available as a Word document, .docx format). Differential Metabolites: Data on the differential metabolites among the various *Taxillus chinensis* groups are provided in the Supplementary File named “Supplementary Tables” (available as a Word document, .docx format).

## Supplementary Materials

The following supporting information can be downloaded at: https://www.mdpi.com/article/10.3390/metabo16070446/s1. Tables regarding the similarity of *Taxillus chinensis* fingerprints and the recovery rates from method validation are included in the supplementary file named “Supplementary Tables” and are referenced in the sections “Fingerprint Establishment and Similarity Evaluation” and “Recovery Test” (available as a Word document, .docx format). Based on the in-house metabolic database, qualitative and quantitative mass spectrometry analyses of the metabolites in the samples were conducted. The multi-peak chromatogram from the Multiple Reaction Monitoring (MRM) mode illustrates the substances detectable within the samples, where each differently colored chromatographic peak represents an individual detected metabolite. Characteristic ions for each substance were screened using a triple quadrupole mass spectrometer, and their signal intensities (CPS) were acquired by the detector. The raw mass spectrometry data files were processed using MultiQuant software 3.0.3 for the integration and correction of chromatographic peaks. The area of each chromatographic peak represents the relative content of its corresponding substance. Finally, all integrated peak area data were exported and saved. To compare the differences in the content of each individual metabolite across different samples among all detected metabolites, the chromatographic peaks for each metabolite detected in various samples were corrected and aligned based on their retention times and peak shapes. This process ensures the accuracy of both qualitative and quantitative analyses. The figure below demonstrates the integral correction results from the quantitative analysis of randomly selected metabolites across different samples. The horizontal axis indicates the retention time (min) of the metabolite detection, while the vertical axis represents the ion current intensity (cps) for a specific metabolite ion. (For more details, please refer to Supplementary Figures S1 and S2 in the file “Supplementary Analytical Methods”).

## References

[1] National Pharmacopoeia Committee (2025). Pharmacopoeia of the People’s Republic of China. Part I.

[2] Zhu K., Lu D., Pei H., Zhao M., Li Y. (2010). Investigation on the Distribution and Host Status of *Taxillus chinensis* in Guangxi. Guangxi J. Tradit. Chin. Med..

[3] Liu C. (2019). Quality Marker (Q-Marker): Improving Quality Standards and Quality Control Theory of Traditional Chinese Medicine and Promoting Scientific Development of the TCM Industry. Chin. Tradit. Herb. Drugs.

[4] Tian S., Liao C., Zhou Z., Tang Q., Li F., Song S., Hu S., Xu Y. (2022). Research Progress and Prospects of Plant Metabolomics in Quality Evaluation of Medicinal Materials. Acta Pharm. Sin..

[5] Li Z.-Y., Cui Y.-F., Qin X.-M. (2018). Challenge of quality evaluation of traditional Chinese medicinal materials and application progress on metabolomic approach in its quality valuation. Zhongcaoyao.

[6] Li L., Teng J., Zhu Y., Xie F., Hou J., Ling Y., Zhu H. (2021). Metabolomics Study of Flavonoids of *Taxillus chinensis* on Different Hosts Using UPLC-ESI-MS/MS. Molecules.

[7] Yuan J., Wu N., Cai Z., Chen C., Zhou Y., Chen H., Xue J., Liu X., Wang W., Cheng J. (2023). Metabolite Profiling and Transcriptome Analysis Explain the Difference in Accumulation of Bioactive Constituents in Taxilli Herba from Two Hosts. Genes.

[8] Li L.-Z. (2021). Study on the Effect of Host on the Medicinal Properties and Substantial Composition of Taxilli Herba.

[9] Zhuo Y., Wang M., Zou L., Tang H., He H. (2025). Evaluation of the Quality of *Taxillus chinensis* Based on HPLC Fingerprint and Multi-component Quantification. J. Chin. Med. Mater..

[10] Pant P., Pandey S., Dall’Acqua S. (2021). The influence of environmental conditions on secondary metabolites in medicinal plants: A literature review. Chem. Biodivers..

[11] Huang L., Guo L. (2007). Accumulation of Secondary Metabolites under Environmental Stress and Formation of Genuine Medicinal Materials. China J. Chin. Mater. Med..

[12] Yang Q., Teng J., Wei H., Li Z., Liang J., Fan L., Zhu Y., Wang X., Li L. (2026). Exploring the Potential of UHPLC-Q-Orbitrap HRMS-Based Untargeted Metabolomics for Discriminating *Spatholobus suberectus* Dunn from Its Adulterants. Biomed. Chromatogr. BMC.

[13] Jin S.-Y., Cai X., Deng Y., Sun B.-H., Feng J.-C., Sun G.-X., Tan H.-L., Sun W.-Y. (2025). Multidimensional fingerprint quality evaluation and main component analysis of Radix Fici Simplicissimae. Microchem. J..

[14] Yang F., Zhang Y., Yu Y., Gao Q., Sun G. (2019). Quality assessment of licorice extract powder through geometric linear quantified fingerprint method combined with multicomponent quantification and chemometric analysis. Microchem. J..

[15] Jin Z., Zhang X., Zhang Q., Qiao L., Zhang W. (2023). Reconstruction of Materia Medica—*Taxillus chinensis*. Jilin J. Tradit. Chin. Med..

[16] Pei H., Huang J., Zhu K., Su B., Zhao M., Zhang M. (2016). Industrialization Development Prospect of Famous and Precious Genuine Medicinal Material *Taxillus chinensis*. China Pharm..

[17] Li L. (2022). Quality Study of *Taxillus chinensis* Resources from Guangxi Based on Metabolomics. Doctoral Dissertation.

[18] Ninkuu V., Aluko O.O., Yan J., Zeng H., Liu G., Zhao J., Li H., Chen S., Dakora F.D. (2025). Phenylpropanoids metabolism: Recent insight into stress tolerance and plant development cues. Front. Plant Sci..

[19] Zagorcheva T., Teofanova D., Odjakova M., Li J., Zagorchev L. (2026). Pharmacological Properties of Parasitic Plants: Current Evidence and the Role of Parasitic Lifestyle. Plants.

[20] Meng X., Lv N., Wang X., Zhou Q., Zhang X., Zhang X., Zhang Z., Liu L., Shen T. (2025). Molecular Mechanism of *Cuscuta* Haustorium Specialization Inferences from Transcriptome and Metabolome Analysis. Metabolites.

[21] Maeda H., Dudareva N. (2012). The shikimate pathway and aromatic amino Acid biosynthesis in plants. Annu. Rev. Plant Biol..

[22] Wang H., Guan J., Feng J., Niu Y., Wang X., Cui Y. (2018). Effect of Total Flavonoids of *Taxillus chinensis* on Adjuvant Arthritis Model in Rats. World Chin. Med..

[23] Zhao H., Wang J., Cui Y., Feng J., Wang X. (2016). Study on Efficacious Substances and Meridian Tropism of *Taxillus chinensis* for Tonifying Liver and Kidney and Strengthening Tendons and Bones Based on “Syndrome-Effect-Biological Sample Analysis” Method. World Sci. Technol.-Mod. Tradit. Chin. Med..

[24] Zhou D., Chai Z., Ru M., Huang F., Zhang X., Guo M., Li Y. (2025). Analysis of the impact of host plants on the quality of *Taxilli herba* based on widely targeted metabolomics. China J. Chin. Mater. Med..

[25] Li L., Guan J., Feng J., Ma K., Cui Y. (2018). Study on Material Basis for Dispelling Wind-Dampness and Meridian Tropism of Total Flavonoids from *Taxillus chinensis* Based on “Disease-Syndrome-Effect-Biological Sample Analysis” Method. China J. Tradit. Chin. Med. Pharm..

[26] Xiong W., Yuan Z., Wang T., Wu S., Xiong Y., Yao Y., Yang Y., Wu H. (2021). Quercitrin Attenuates Acetaminophen-Induced Acute Liver Injury by Maintaining Mitochondrial Complex I Activity. Front. Pharmacol..

[27] Guo H., Yin W., Zou Z., Zhang C., Sun M., Min L., Yang L., Kong L. (2021). Quercitrin alleviates cartilage extracellular matrix degradation and delays ACLT rat osteoarthritis development: An in vivo and in vitro study. J. Adv. Res..

[28] Kim C.H., Kim J.-E., Song Y.-J. (2020). Antiviral Activities of Quercetin and Isoquercitrin Against Human Herpesviruses. Molecules.

[29] Habiburrahman M., Sutopo S., Rahadiani N. (2023). The plausible use of mango (*Mangifera indica*) peel isoquercitrin as adjuvant therapy for colorectal cancer: Translating research from bench to bedside. Indones. J. Gastroenterol. Hepatol. Dig. Endosc..

[30] Kahkeshani N., Farzaei F., Fotouhi M., Alavi S.S., Bahramsoltani R., Naseri R., Momtaz S., Abbasabadi Z., Rahimi R., Farzaei M.H. (2019). Pharmacological effects of gallic acid in health and disease: A mechanistic review. Iran. J. Basic Med. Sci..

[31] Kubicova L., Bachmann G., Weckwerth W., Chobot V. (2022). (±)-Catechin—A mass-spectrometry-based exploration coordination complex formation with Fe^II^ and Fe^III^. Cells.

[32] Dash J.R., Pattnaik G., Ghosh G., Rath G., Kar B. (2023). Protective effect of epicatechin in diabetic-induced peripheral neuropathy: A review. J. Appl. Pharm. Sci..

[33] Rozza A.L., Hiruma-Lima C.A., Tanimoto A., Pellizzon C.H. (2012). Morphologic and pharmacological investigations in the epicatechin gastroprotective effect. *Evid.-Based Complement*. Altern. Med. eCAM.

[34] German I.J.S., Pomini K.T., Andreo J.C., Shindo J.V.T.C., de Castro M.V.M., Detregiachi C.R.P., Araújo A.C., Guiguer E.L., Laurindo L.F., Bueno P.C.d.S. (2024). New Trends to Treat Muscular Atrophy: A Systematic Review of Epicatechin. Nutrients.

[35] Shay J., Elbaz H.A., Lee I., Zielske S.P., Malek M.H., Hüttemann M. (2015). Molecular Mechanisms and Therapeutic Effects of (−)-Epicatechin and Other Polyphenols in Cancer, Inflammation, Diabetes, and Neurodegeneration. Oxidative Med. Cell. Longev..

[36] Xu S., Chen S., Xia W., Sui H., Fu X. (2022). Hyperoside: A Review of Its Structure, Synthesis, Pharmacology, Pharmacokinetics and Toxicity. Molecules.

