# Supplementary material for: Comprehensive Phytochemical Characterization and Quality Evaluation of Taxillus chinensis via Integrated Widely Targeted Metabolomics, HPLC Fingerprinting, and Multi-Component Quantification
Source: Metabolites. 2026 Jun 25;16(7):446. doi: 10.3390/metabo16070446 (PMC13414450; doi:10.3390/metabo16070446)
Supplement: Supplementary file 1 [file metabolites-16-00446-s001.zip › OPLS-DA analysis.pdf]

OPLS-DA analysis

In the OPLS-DA model comparing samples from different origins hosted by *Morus* (CSJ, NSJ, WSJ, and YSJ), the parameters were $R^2X = 0.62$, $R^2Y = 0.999$, and $Q^2 = 0.844$ (Figure S1 A and B). For the model comparing samples from Wuzhou parasitizing different hosts (WSJ, WFJ, and WSO), the parameters were $R^2X = 0.433$, $R^2Y = 0.989$, and $Q^2 = 0.864$. The Q^2 values for both models exceeded 0.8, indicating robust predictive capability and the absence of overfitting.(Figure S1 C and D)

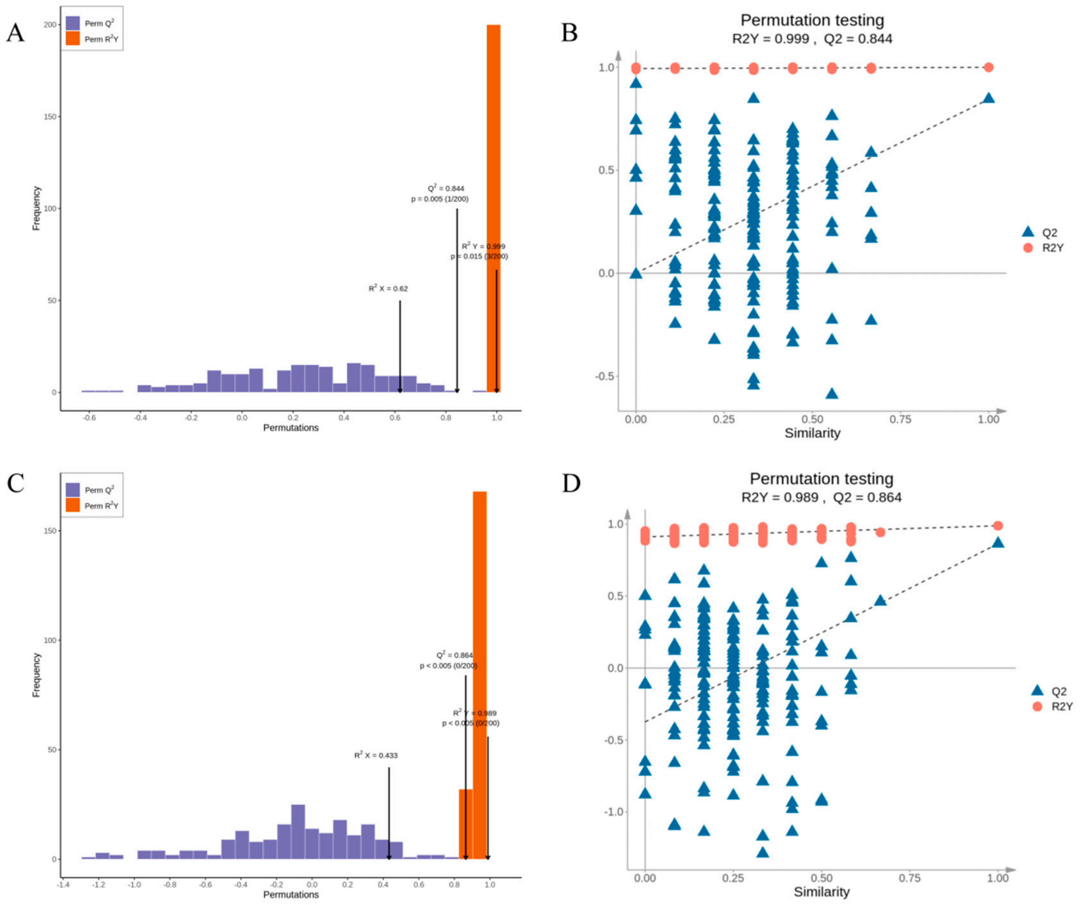

Figure S1 A: OPLS-DA model validation plot for YSJ vs NSJ vs WSJ vs CSJ; Figure B: OPLS-DA permutation test score plot for YSJ vs NSJ vs WSJ vs CSJ, Figure C: OPLS-DA model validation plot for WSJ vs WFJ vs WSO; Figure D: OPLS-DA permutation test score plot for WSJ vs WFJ vs WSO.
