# Supplementary material for: Comprehensive Phytochemical Characterization and Quality Evaluation of Taxillus chinensis via Integrated Widely Targeted Metabolomics, HPLC Fingerprinting, and Multi-Component Quantification
Source: Metabolites. 2026 Jun 25;16(7):446. doi: 10.3390/metabo16070446 (PMC13414450; doi:10.3390/metabo16070446)
Supplement: Supplementary file 1 [file metabolites-16-00446-s001.zip › Supplementary Tables.pdf]

Table S1 The number and trend of differential metabolites in *Taxillus chinensis* hosted on *Morus alba* from different producing areas

Compound class	Amount of metabolites Amount of metabolites												Total
	CSJ vs WSJ		CSJ vs YSJ		NSJ vs WSJ		NSJ vs YSJ		NSJ vs CSJ		YSJ vs WSJ		
	Up	Down	Up	Down	Up	Down	Up	Down	Up	Down	Up	Down	
Flavonoids	29	57	31	31	14	20	42	7	42	15	18	44	350
Phenolic acids	36	24	26	13	27	13	48	15	17	11	28	46	304
Alkaloids	38	0	18	5	23	4	10	7	6	22	22	6	161
Lignans and Coumarins	32	6	18	2	21	5	19	11	3	11	25	16	169
Quinones	1	1	1	1	0	0	1	0	0	1	0	0	6
Tannins	5	0	7	0	1	1	7	2	1	2	2	7	35
Terpenoids	16	17	13	4	12	6	12	8	12	17	13	19	149
Steroids	0	0	0	0	0	0	0	0	0	1	0	0	1
Others	31	8	16	3	28	5	21	11	18	8	18	19	186
Total	188	113	130	59	126	54	160	61	97	90	127	156	1361
	301		189		180		221		187		283		

Table S2 The number and trend of differential metabolites in *Taxillus chinensis* from the same producing area but hosted on different hosts.

Compound class	Amount of metabolites Amount of metabolites						Total
	WFJ vs WSJ		WSO vs WSJ		WFJ vs WSO		
	Up	Down	Up	Down	Up	Down	
Flavonoids	38	32	27	11	27	32	167
Phenolic acids	20	28	14	7	15	37	121

Compound class	Amount of metabolites Amount of metabolites						Total
	WFJ vs WSJ		WSO vs WSJ		WFJ vs WSO		
	Up	Down	Up	Down	Up	Down	
Alkaloids	24	10	19	1	9	15	78
Lignans and Coumarins	36	8	13	0	23	8	88
Quinones	0	1	0	0	0	1	2
Tannins	4	1	1	1	2	7	16
Terpenoids	16	13	5	2	9	19	64
Steroids	0	0	0	0	0	0	0
Others	17	13	9	5	13	19	76
Total	152	109	88	27	98	138	612
	261		115		236		

Table S3 Top 10 Differential Metabolites of CSJ vs. WSJ

Compounds	Class I	Formula	VIP	log2FC	Type
Cyanidin-3-O-galactoside	Flavonoids	C21H21O11+	1.461755168	-8.601490858	Down
Cyanidin-3-O-glucoside	Flavonoids	C21H21O11+	1.459887235	-8.317733642	Down
Cyanidin 3-(2"-galloylglucoside)	Flavonoids	C28H25O15+	1.470812732	-7.177479869	Down
Chisopanin C	Terpenoids	C33H52O6	1.035800974	-5.473501558	Down
2-(3,4-Dihydroxybenzoyloxy)-4,6-Dihydroxyphenylacetic Acid	Phenolic acids	C15H12O8	1.461521146	-4.746092636	Down
1-Methylnicotinamide	Alkaloids	C7H9N2O	1.438275959	4.620203975	up
Trigonelline	Alkaloids	C7H7NO2	1.436038731	4.650512298	up

Compounds	Class I	Formula	VIP	log2FC	Type
Aviculin	Lignans and Coumarins	C26H34O 10	1.3805 99482	4.765458 472	up
Salicyolylsalicortin	Phenolic acids	C27H28O 12	1.2682 17817	4.782810 858	up
Dehydrodiconiferylalcohol-9'-O-glucoside	Lignans and Coumarins	C26H32O 11	1.4397 36736	5.059704 661	up

Table S4 Top 10 Differential Metabolites of CSJ vs. YSJ

Compounds	Class I	Formula	VIP	log2FC	Type
Cyanidin-3-O-galactoside	Flavonoids	C21H21 O11+	1.55963 4296	-3.09152 3279	down
Cyanidin-3-O-glucoside	Flavonoids	C21H21 O11+	1.53148 519	-3.03112 1807	down
Scutellarin	Flavonoids	C21H18 O12	1.38065 7658	-2.94128 4602	down
Pyridine-4-formyl-O-β-D-glucopyranoside	Alkaloids	C12H15 NO7	1.35113 7805	-2.92175 0804	down
Cyanidin 3-(2''-galloylglucoside)	Flavonoids	C28H25 O15+	1.11263 9665	-2.81264 0027	down
Salicyolylsalicortin	Phenolic acids	C27H28 O12	1.30141 3127	4.259201 772	up
Isohemiphloin	Flavonoids	C21H22 O10	1.54963 7239	4.259401 183	up
p-Coumaraldehyde	Others	C9H8O2	1.56319 559	4.280942 944	up
Pterosupin	Flavonoids	C21H24 O10	1.56771 1382	4.394778 86	up
4-C-Glucose-1,3,6-trihydroxy-7-methoxyxanthone	Flavonoids	C20H20 O11	1.56803 2503	4.983876 294	up

Table S5 Top 10 Differential Metabolites of NSJ vs. CSJ

Compounds	Class I	Formula	VIP	log2FC	Type
Dehydrodiconiferylalcohol-9'-O-glucoside	Lignans and Coumarins	C26H32O11	1.637704546	-4.481541803	down
Salicyolylsalicortin	Phenolic acids	C27H28O12	1.320740291	-3.909772357	down
Methyl caffeate	Phenolic acids	C10H10O4	1.376710253	-3.748969405	down
(3R)-Hydroxy-Beta-Ionone Malonylglucoside	Terpenoids	C22H32O10	1.639890129	-3.474751893	down
3-Oxo-Alpha-Ionol 3'-(6"-Malonyl)Glucoside	Terpenoids	C22H32O10	1.651831849	-3.418973814	down
Pyridine-4-formyl-O-β-D-glucopyranoside	Alkaloids	C12H15NO7	1.450601131	2.987955953	up
Cyanidin-3-O-glucoside	Flavonoids	C21H21O11+	1.665171075	3.285713964	up
(4β,10E)-6α,15-Dihydroxy-8β-(angeloyloxy)-14-oxogermacra-1(10),11(13)-diene-12-oic acid-12,6-lactone	Terpenoids	C20H26O6	1.622295728	3.351717016	up
Cyanidin-3-O-galactoside	Flavonoids	C21H21O11+	1.67065947	3.611381126	up
Chisopanin C	Terpenoids	C33H52O6	1.679273341	7.049708229	up

Table S6 Top 10 Differential Metabolites of NSJ vs. WSJ

Compounds	Class I	Formula	VIP	log2FC	Type
Cyanidin 3-(2"-galloylglucoside)	Flavonoids	C28H25O15+	1.696838243	-5.955087448	down
Cyanidin-3-O-glucoside	Flavonoids	C21H21O11+	1.704051261	-5.032019678	down
Cyanidin-3-O-galactoside	Flavonoids	C21H21O11+	1.709610377	-4.990109733	down
Phyllanemblinin D	Tannins	C27H26	1.45705	-3.77891	down

Compounds	Class I	Formula	VIP	log2FC	Type
		O20	967	6944	
2-O-Caffeoylglucaric Acid	Phenolic acids	C15H16 O11	1.60563 2811	-2.91980 3768	down
Isolariciresinol-9'-O-rhamnoside (Aviculin)	Lignans and Coumarins	C26H34 O10	1.61176 6121	3.778818 858	up
3-Carbamyl-1-methylpyridinium; (1-Methylnicotinamide)	Alkaloids	C7H9N2 O	1.54681 238	4.170538 464	up
4-[(1e)-3-hydroxyprop-1-en-1-yl] -2,6-dimethoxyphenyl(2e)-3-phen ylprop-2-enoate	Lignans and Coumarins	C20H20 O5	1.27022 4891	4.217521 976	up
Trigonelline	Alkaloids	C7H7NO 2	1.54864 4676	4.218838 742	up
3'-p-Coumaroyl-sucrose	Phenolic acids	C21H28 O13	1.73409 2994	5.272607 56	up

Table S7 Top 10 Differential Metabolites of NSJ vs. YSJ

Compounds	Class I	Formula	VIP	log2FC	Type
Methyl caffeate	Phenolic acids	C10H10 O4	1.50205 0503	-4.04832 7381	down
Tricin-5-O-Glucoside	Flavonoids	C23H24 O12	1.55550 5186	-3.51976 2629	down
8-Hydroxy-10-hydrosweroside	Others	C16H24 O10	1.54447 5189	-3.25215 1702	down
Matairesinoside	Lignans and Coumarins	C26H32 O11	1.35161 6854	-3.14568 1716	down
3-Oxo-Alpha-Ionol 3'-(6''-Malonyl)Glucoside	Terpenoids	C22H32 O10	1.35579 1263	-2.83892 1654	down
tert-butyl (3S)-3,4-dihydroxy-4-[(4-methox yphenyl)methoxy]butanoate	Phenolic acids	C16H24 O6	1.48034 153	3.485654 414	up
Pterosupin	Flavonoids	C21H24 O10	1.48530 2752	3.668129 105	up
Digalloyl-HHDP-glucose	Tannins	C34H26	1.54817	3.813016	up

Compounds	Class I	Formula	VIP	log2FC	Type
		O22	044	801	
4-C-Glucose-1,3,6-trihydroxy-7-methoxyxanthone	Flavonoids	C20H20 O11	1.47904 2834	3.996474 059	up
Chisopanin C	Terpenoids	C33H52 O6	1.58388 7798	7.049708 229	up

Table S8 Top 10 Differential Metabolites of YSJ vs. WSJ

Compounds	Class I	Formula	VIP	log2FC	Type
Cyanidin-3-O-galactoside	Flavonoids	C21H21 O11+	1.46825 3459	-5.50996 758	down
Chisopanin C	Terpenoids	C33H52 O6	1.05045 6785	-5.47350 1558	down
Cyanidin-3-O-glucoside	Flavonoids	C21H21 O11+	1.45679 3206	-5.28661 1835	down
Cyanidin 3-(2"-galloylglucoside)	Flavonoids	C28H25 O15+	1.34635 1048	-4.36483 9842	down
Phyllanemblinin D	Tannins	C27H26 O20	1.26562 7887	-3.95367 8329	down
Epigallocatechin-(4beta->8)-cate chin	Tannins	C30H26 O13	1.47547 3844	3.879710 037	up
Methyl caffeate	Phenolic acids	C10H10 O4	1.38395 5852	3.885494 958	up
3-Carbamyl-1-methylpyridinium; (1-Methylnicotinamide)	Alkaloids	C7H9N2 O	1.43997 4818	3.950983 302	up
Trigonelline	Alkaloids	C7H7NO 2	1.43535 6923	3.976876 984	up
2-Acetyl-3-hydroxyphenyl-1-O-g lucoside	Others	C15H20 O7	1.42515 2265	4.487939 87	up

Table S9 Top 10 Differential Metabolites of WFJ vs. WSJ

Compounds	Class I	Formula	VIP	Log2FC	Type
1-Deoxynojirimycin	Alkaloids	C6H13NO4	1.552617921	-9.509945508	down
6-acetyldelpheline	Alkaloids	C27H41NO7	1.556999084	-8.256989909	down
D-Linalool 3-(6"-Malonylglucoside)	Terpenoids	C19H30O9	1.541104002	-6.612044118	down
Phyllanemblinin D	Tannins	C27H26O20	1.4169917	-5.318928718	down
Pinoresinol-4-O-(6"-acetyl)glucoside	Lignans and Coumarins	C28H34O12	1.499425963	-5.201759067	down
3'-p-Coumaroyl-sucrose	Phenolic acids	C21H28O13	1.559200418	5.963944052	up
Pedaliin	Flavonoids	C22H22O12	1.549150442	6.146225796	up
Loganic acid	Terpenoids	C16H24O10	1.512405089	6.63328419	up
Clemaphenol A	Lignans and Coumarins	C20H22O6	1.554855654	7.744488328	up
Geniposidic acid	Terpenoids	C16H22O10	1.559925772	7.763463232	up

Table S10 Top 10 Differential Metabolites of WFJ vs. WSO

Compounds	Class I	Formula	VIP	Log2FC	Type
1-Deoxynojirimycin	Alkaloids	C6H13NO4	1.552617921	-9.509945508	down
6-acetyldelpheline	Alkaloids	C27H41NO7	1.556999084	-8.256989909	down
D-Linalool 3-(6"-Malonylglucoside)	Terpenoids	C19H30O9	1.541104002	-6.612044118	down
Phyllanemblinin D	Tannins	C27H26O20	1.4169917	-5.318928718	down

Compounds	Class I	Formula	VIP	Log2FC	Type
Pinoresinol-4-O-(6''-acetyl)glucoside	Lignans and Coumarins	C28H34O12	1.499425963	-5.201759067	down
3'-p-Coumaroyl-sucrose	Phenolic acids	C21H28O13	1.559200418	5.963944052	up
Pedaliin	Flavonoids	C22H22O12	1.549150442	6.146225796	up
Loganic acid	Terpenoids	C16H24O10	1.512405089	6.63328419	up
Clemaphenol A	Lignans and Coumarins	C20H22O6	1.554855654	7.744488328	up
Geniposidic acid	Terpenoids	C16H22O10	1.559925772	7.763463232	up

Table S11 Top 10 Differential Metabolites of WSO vs. WSJ

Compounds	Class I	Formula	VIP	Log2FC	Type
Cyanidin-3-O-galactoside	Flavonoids	C21H21O11+	1.676898989	-5.24398756	down
Cyanidin-3-O-glucoside	Flavonoids	C21H21O11+	1.680374811	-5.176475396	down
Cyanidin 3-(2''-galloylglucoside)	Flavonoids	C28H25O15+	1.592333399	-4.274911765	down
Phyllanemblinin D	Tannins	C27H26O20	1.076027538	-2.29664174	down
5,7,3',4',5'-pentahydroxyflavan glucoside	Flavonoids	C21H24O11	1.152339579	-1.933097721	down
3-(2'-Hydroxyl-phenyl)-4-(3H)-quinazolone	Alkaloids	C15H10N2O3	1.725883888	3.088224043	up
Cyanidin-3-O-(6''-O-p-hydroxybenzoyl)sophoroside-5-O-glucoside	Flavonoids	C40H45O23+	1.729012466	3.240376617	up
4-C-Glucose-1,3,6-trihydroxy-7-methoxyxanthone	Flavonoids	C20H20O11	1.508760478	3.382088196	up

Compounds	Class I	Formula	VIP	Log2FC	Type
Epigallocatechin-(4beta->8)-catechin	Tannins	C30H26O13	1.722692304	3.42199451	up
Clemaphenol A	Lignans and Coumarins	C20H22O6	1.699495859	3.724972083	up

Table S12 Similarity evaluation results of 20 batches of Taxillus chinensis

No.	Similarity	No.	Similarity	No.	Similarity	No.	Similarity
S1	0.989	S6	0.948	S11	0.973	S16	0.972
S2	0.919	S7	0.987	S12	0.988	S17	0.98
S3	0.977	S8	0.989	S13	0.948	S18	0.993
S4	0.929	S9	0.977	S14	0.97	S19	0.99
S5	0.955	S10	0.989	S15	0.987	S20	0.989

Table S13 Spiking Recovery Test Results (*n*=6)

Component	Original amount（μg）	Added amount（μg）	Measured amount（μg）	Recovery rate（%）	Average value（%）	RSD（%）
Gallic acid	1.7233	1.6000	3.3301	100.43%	101.12%	0.99%
	1.7077	1.6000	3.3181	100.65%		
	1.7242	1.6000	3.3192	99.69%		
	1.7209	1.6000	3.3495	101.79%		
	1.6989	1.6000	3.3314	102.03%		
	1.6937	1.6000	3.3278	102.13%		
Catechin	44.5714	45.0000	90.8165	102.77%	102.57%	0.22%
	45.8037	45.0000	90.9862	100.41%		
	45.3359	45.0000	90.9127	101.28%		
	45.0507	45.0000	91.3235	102.83%		
	44.1039	45.0000	90.9379	104.08%		

	44.3891	45.0000	91.2230	104.08%		
Epicatechin	5.8833	6.0000	11.6399	95.94%	99.19%	1.93%
	6.0011	6.0000	12.0294	100.47%		
	5.9933	6.0000	12.0572	101.07%		
	6.1346	6.0000	12.0160	98.02%		
	6.0374	6.0000	12.0566	100.32%		
	5.9736	6.0000	11.9315	99.30%		
Hyperoside	21.2599	21.0000	42.1925	99.68%	97.98%	1.52%
	21.2483	21.0000	41.2958	95.46%		
	21.3607	21.0000	42.0511	98.53%		
	21.4127	21.0000	42.1450	98.73%		
	21.2888	21.0000	41.9607	98.44%		
	20.9505	21.0000	41.3297	97.04%		
Isoquercitrin	5.0666	5.0000	10.0920	100.51%	97.36%	1.07%
	5.0614	5.0000	9.8675	96.12%		
	4.9866	5.0000	9.9087	98.44%		
	5.0512	5.0000	9.8618	96.21%		
	5.0242	5.0000	9.7871	95.26%		
	5.0956	5.0000	9.9757	97.60%		
Quercitrin	73.5612	73.0000	148.9734	103.30%	102.68%	1.05%
	72.3768	73.0000	148.0109	103.61%		
	73.5842	73.0000	148.6456	102.82%		
	72.1906	73.0000	147.5914	103.29%		
	72.0903	73.0000	146.8668	102.43%		
	73.4000	73.0000	146.8668	100.64%		

Table S14 Content determination results of *Taxillus chinensis* samples

Sample	Gallic acid （mg/g）	Catechin （mg/g）	Epicatechin （mg/g）	Hyperoside （mg/g）	Isoquercitrin （mg/g）	Quercitrin （mg/g）	Total （mg/g）

S1	4.06	45.25	8.35	16.85	7.12	50.98	132.61
S2	9.27	20.97	5.05	27.77	4.12	40.37	107.56
S3	2.65	19.24	2.94	17.27	2.49	112.37	156.97
S4	4.28	21.89	3.15	10.74	2.01	21.62	63.69
S5	8.52	26.13	5.11	13.06	3.06	31.14	87.03
S6	2.50	18.48	4.76	1.23	2.48	128.45	157.90
S7	1.33	46.94	8.24	10.31	6.12	59.45	132.39
S8	2.72	49.33	4.51	14.14	3.63	46.89	121.22
S9	7.85	20.67	4.68	24.99	2.95	54.07	115.21
S10	6.03	23.05	5.04	18.18	5.31	45.38	103.00
S11	14.13	17.91	4.05	42.13	11.85	92.90	182.98
S12	1.77	12.88	2.80	15.84	4.90	68.92	107.11
S13	3.57	18.46	3.02	33.57	3.32	53.64	115.59
S14	5.97	77.09	6.51	15.15	5.17	55.79	165.69
S15	1.35	67.97	6.28	15.99	5.65	65.88	163.12
S16	3.08	83.69	9.10	17.55	5.09	61.15	179.64
S17	2.94	40.08	4.42	8.47	8.50	47.37	111.78
S18	1.54	38.48	4.99	14.69	4.21	70.51	134.43
S19	3.02	42.97	5.34	14.66	4.92	43.92	114.83
S20	2.01	28.49	5.33	16.81	4.89	91.89	149.41
