# Supplementary figures and images for: Comprehensive Phytochemical Characterization and Quality Evaluation of Taxillus chinensis via Integrated Widely Targeted Metabolomics, HPLC Fingerprinting, and Multi-Component Quantification
Source: Metabolites. 2026 Jun 25;16(7):446. doi: 10.3390/metabo16070446 (PMC13414450; doi:10.3390/metabo16070446)

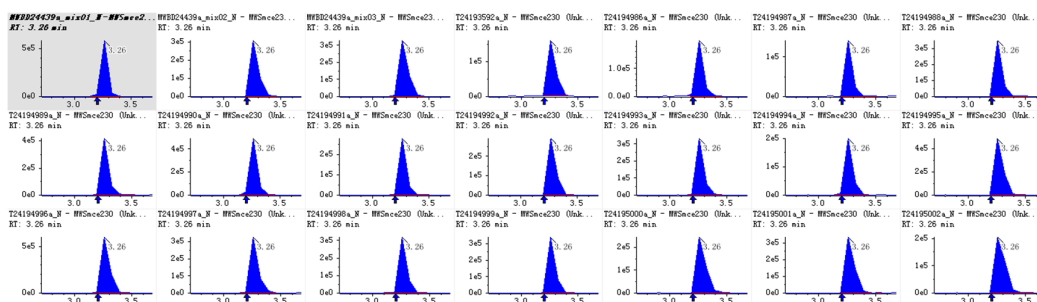

Figure S1 Integral\_correction\_diagram-N

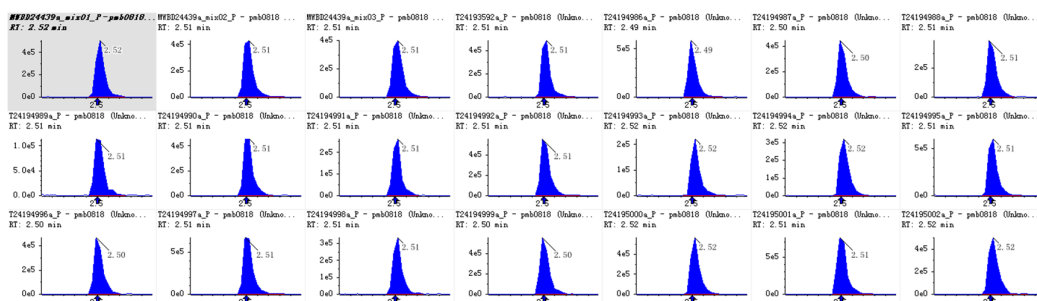

Figure S2 Integral\_correction\_diagram-P

Supplement: Supplementary file 1 [file metabolites-16-00446-s001.zip › Supplementary Analytical Methods.pdf]
